# Study on a Pig Vocalization Classification Method Based on Multi-Feature Fusion

**DOI:** 10.3390/s24020313

**Published:** 2024-01-05

**Authors:** Yuting Hou, Qifeng Li, Zuchao Wang, Tonghai Liu, Yuxiang He, Haiyan Li, Zhiyu Ren, Xiaoli Guo, Gan Yang, Yu Liu, Ligen Yu

**Affiliations:** 1 Research Center of Information Technology, Beijing Academy of Agriculture and Forestry Sciences, Beijing 100097, China; hyt2799009806@163.com (Y.H.); liqf@nercita.org.cn (Q.L.); botanylihaiyan@163.com (H.L.); renzy@nercita.org.cn (Z.R.); 18338786103@163.com (X.G.); yannng1516@163.com (G.Y.); 2 School of Science, China University of Geosciences (Beijing), Beijing 100083, China; wzc@cugb.edu.cn; 3 National Engineering Research Center for Information Technology in Agriculture, Beijing 100097, China; 4 College of Computer and Information Engineering, Tianjin Agricultural University, Tianjin 300384, China; tonghai_1227@126.com (T.L.); heyuxiang@tjau.edu.cn (Y.H.)

**Keywords:** pig vocalization, multi-feature fusion, principal component analysis, classification recognition

## Abstract

To improve the classification of pig vocalization using vocal signals and improve recognition accuracy, a pig vocalization classification method based on multi-feature fusion is proposed in this study. With the typical vocalization of pigs in large-scale breeding houses as the research object, short-time energy, frequency centroid, formant frequency and first-order difference, and Mel frequency cepstral coefficient and first-order difference were extracted as the fusion features. These fusion features were improved using principal component analysis. A pig vocalization classification model with a BP neural network optimized based on the genetic algorithm was constructed. The results showed that using the improved features to recognize pig grunting, squealing, and coughing, the average recognition accuracy was 93.2%; the recognition precisions were 87.9%, 98.1%, and 92.7%, respectively, with an average of 92.9%; and the recognition recalls were 92.0%, 99.1%, and 87.4%, respectively, with an average of 92.8%, which indicated that the proposed pig vocalization classification method had good recognition precision and recall, and could provide a reference for pig vocalization information feedback and automatic recognition.

## 1. Introduction

In many countries, pigs are the main source of meat for people, and pork is an important part of livestock products and food composition and has considerable economic value [1]. By 2021, the total pork output of China had reached 52.959 million tons, which accounted for the largest proportion of total meat production, that is, approximately 58.9%. The huge demand for pork has accelerated the development of large-scale pig farming and promoted higher requirements for intensive and specialized modern pig breeding technology [2]. With the transformation of the pig breeding mode, the health status and welfare level of pigs have also attracted increasing attention [3]. Particularly in large-scale breeding houses, because of the high feeding density, it is difficult for farmers to take good care of each pig and detect pig abnormalities in a timely manner based on manual inspection alone [4]. A delay in the treatment of sick pigs may cause heavy production losses. Vocalization is an important way for pigs to transmit real-time health information to greatly improve the efficiency of sick pig evaluation and environmental regulation and promote healthy and efficient pig breeding [5,6].

At present, studies on pig vocalization recognition have mainly focused on the classification of pig voices. A large number of studies have been conducted around the features and classification models of vocal signals and achieved good results. In most studies, Mel frequency cepstral coefficient (MFCC) was frequently used as the key acoustic feature in animal sound category classification and abnormal recognition [7]. In addition, frequency and time domain features, such as root mean square (RMS) and power spectral density (PSD), were also considered as the key features in sound classification [8]. Chung et al. [9] used the support vector data description and sparse representation classifier as the early abnormal monitor and respiratory disease classifier, respectively, by extracting the MFCC. The results showed that the method could be used to accurately monitor pig diseases (94% of monitoring accuracy and 91% of classification accuracy). Studies found there are obvious differences in the time domain and frequency domain features of different types of pig vocalizations [10]. Exadaktylos et al. [11] studied the frequency features of coughing vocalization in sick pigs using power spectral density (PSD) and classified the vocalization, and the accuracy of coughing vocalization recognition was 82%. Xu et al. [12] extracted the vocal PSD feature as the clustering center and identified the coughing and squealing vocalizations of pigs; the overall recognition accuracies were approximately 83.4% and 83.1%, respectively. However, sound data often show poor robustness when their signal-to-noise ratio is low, due to their non-stationary characteristic [7]. Additionally, sounds generally contain multiple acoustic features; it is difficult to further improve sound category classification accuracies by only relying on a single feature [13], especially under real-life production conditions, which restricts certain kinds of classification for acoustic features. Fusion strategies provide a new direction for boosting the accuracy of pig cough sound recognition [7].

Regarding feature fusion, Li et al. [14] combined short-time energy with time domain features and the MFCC dimensionality with frequency domain features, and they further reduced the dimensionality using PCA to construct a deep belief network pig coughing vocalization recognition model fine-tuned by a BP neural network. The recognition rate of pig coughing vocalization was improved and reached 95.8% in the optimal group, which was higher than the results analyzing from single feature [9,11,12,14]. In addition, it was found there were a lot of acoustic features showing up differently among different sound categories. The RMS value of a non-infectious pig cough was higher than that of an infectious pig cough, and there were also significant differences in the duration and short-term energy (STE) of coughing vocalization in healthy pigs and pigs with respiratory diseases [15,16]. Researchers have found that there is a significant difference between the mean value of the formant frequency vocalization of pigs in a normal state and those in an abnormal state. When the mean value of the formant frequency vocalization is lower than 2671.99 Hz and the signal duration is less than 0.28 s, piglets are in a normal state; otherwise, they are in an abnormal state [15,16]. The in-depth clarification of the features of each type of vocalization will be conducive to vocalization classification and vocalization information extraction. However, it is not better to introduce more parameters into classification algorithm, more parameters mean more noise, which will affect the classification performance [17]. Wang et al. [18] reduced the dimensionality of the MFCC features of piglet coughing vocalization using principal component analysis (PCA), the input features were reduced to 13 from 24, and the accuracy achieved 95% using relatively mature and simple support vector machine algorithms. The sound of pig is one of its important pieces of physical information that closely reflect its growth status and health condition; different sound categories are considered as bases for judging the stress state of pigs [19]. In addition to coughing, typical pig sounds include grunting and squealing. In current research on the classification and recognition of abnormal voices in pigs, researchers mainly focus on coughing vocalization, and only few studies focus on the classification and monitoring of various sound types of pigs in large-scale breeding houses, making a lack of the effective mining of the vocal information of pigs, which has seriously weakened the accuracy of vocalization information in reflecting the health condition and breeding environment of pigs. Yu et al. [20] developed a genetic algorithm optimized BP neural network with multi-feature fusion to successfully recognize the typical calls of laying hens, such as egg laying, singing, feeding, and screeching. Although the audio characteristics of pigs are different from those of hens, this study still gives us a good idea to classify and recognize pig sounds using a relatively mature and easy-to-use method.

With the development of signal processing technology, machine learning algorithms have been gradually applied to the field of pig sound categories classification. In this study, the main objective was to develop a vocalization classification model based on multi-feature fusion to classify and identify pig grunting, squealing, and coughing. The sub-objectives were (1) to evaluate the effect of a comprehensive evaluation score as a newly introduced feature on pig sound classification and (2) to compare the influence of different dimensions of features on the recognition effect of the model.

## 2. Materials and Methods

**Figure 1:**
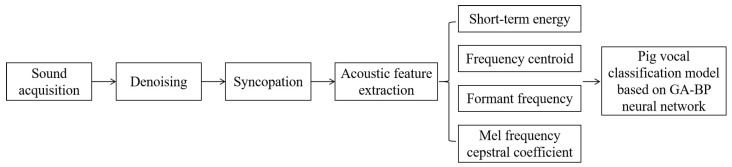
Procedure for pig sound category classification.

In this study, sound data were collected using acoustic equipment during the normal production activities of the pigs and had no impact on the life and normal production activities of the pigs. Then, the data were pre-processed by denoising and syncopating for acoustic features extraction. The extracted features contained STE, frequency centroid (FC), formant frequency (FF), and MFCC. Finally, a three-layer BP neural network was selected to construct the pig vocalization classification model. The process is shown in Figure 1.

### 2.1. Data Collection and Processing

#### 2.1.1. Collection of Pigs’ Vocal Data

**Figure 2:**
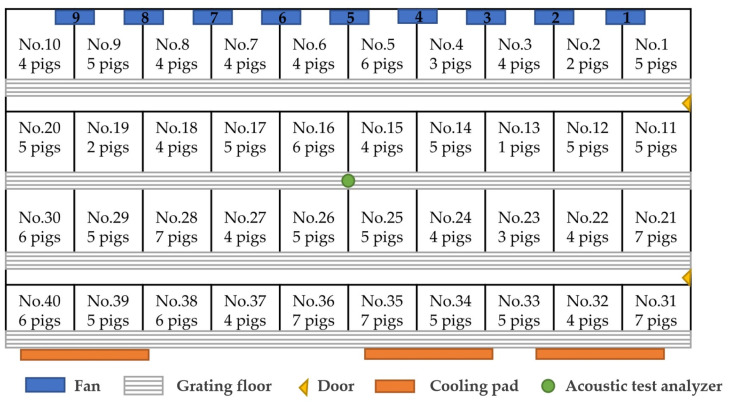
Plan of the pig house.

The study was conducted in Unit 04, Building 6, Shiwan Finishing Pig House of Zhejiang Huateng Animal Husbandry Co., Ltd. located in Tongxiang city of China from 20 October 2021 to 20 November 2021. The test objects were 189 Danish Landraces in the fattening stage. The plan schematic diagram of the pig house is shown in Figure 2.

The pigs’ vocalization data were collected using an acoustic test analyzer (BK 2270-S-C, Hottinger Brüel & Kjær, Nærum, Denmark), which was equipped with a 4189 free-field microphone (sampling frequency: 44.1 kHz; 16-bit resolution; and single channel) and a data logging software (BZ-7226, Hottinger Brüel & Kjær, Nærum, Denmark). The vocalization data collection device was installed at the geometric center point of the house at a height of 2.2 m above the ground. During the test, vocalization data were continuously collected and stored every 10 min, and the storage format was .wav. Thus, the vocal dataset *A_i_* in the Danish Landrace breeding house during the fattening period was obtained.

#### 2.1.2. Denoising of Pig Vocalization

**Figure 3:**
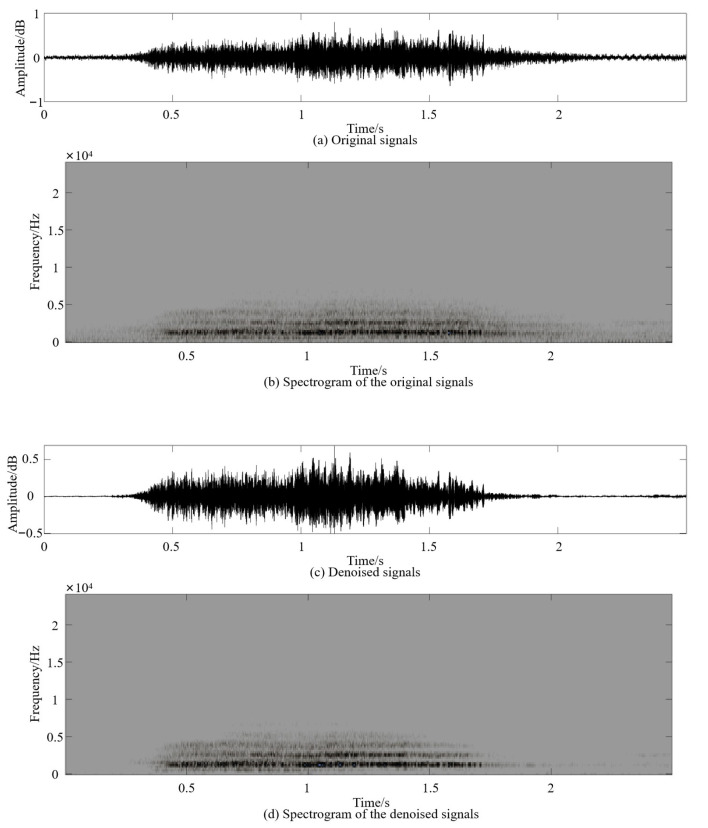
Comparison of the effects before and after pig vocal denoising.

A subset of the vocal dataset *A_i_* generated from 2 November 2021 to 6 November 2021 was selected and denoised using vocal processing software (Adobe Audition CC 2018, Adobe Systems Incorporated, CA, USA) and the adaptive noise reduction method [20]. In this process, the noise reduction amplitude was set to 20 dB, the noise amount was 80%, and the signal threshold was 3 dB. Thus, the pig vocal dataset *B_i_* was obtained. Figure 3 shows a comparison of the effects before and after vocal data denoising in the pig barn. Noise was effectively eliminated and there was no noticeable distortion in the signals.

#### 2.1.3. Syncopation of Pig Vocalization

**Figure 4:**
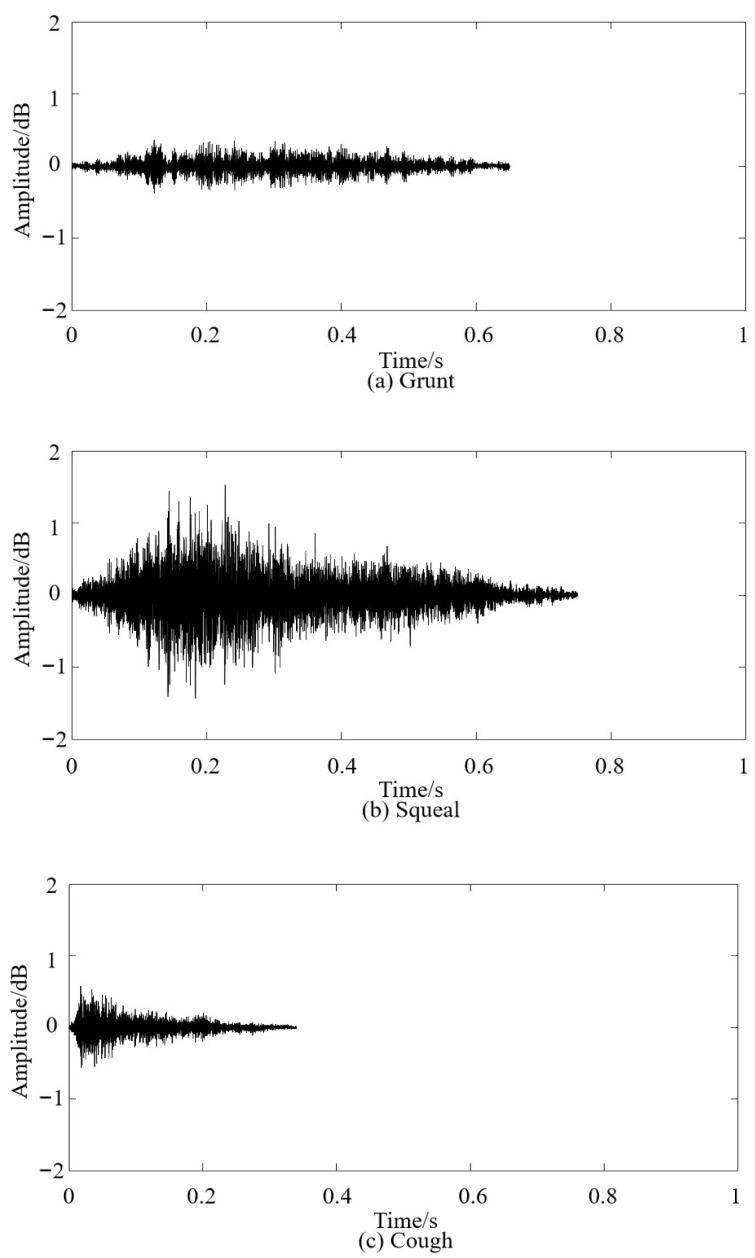
Waveforms of various types of pig vocalization.

The pig vocal dataset *B_i_* was syncopated using vocal processing software, with syllables and phonetic sequences as the units. The typical vocalizations of pigs in the large-scale breeding mode were counted using manual interpretation. A total of 939 clear and non-overlapping pig vocal clips *C_i_* were obtained, including 291 grunts (30.99%), 357 squeals (38.02%), and 291 coughs (30.99%). The waveforms of the three types of pig vocalization are shown in Figure 4.

### 2.2. Feature Extraction and Calculation

To obtain the spectral feature information in the pig vocal signals as comprehensively and fully as possible, the typical features of the time domain and frequency domain during pig vocalization were calculated using vocal processing software. The vocal clip *C_i_* was obtained using vocal preprocessing, and STE, frequency centroid (FC), formant frequency (FF), and MFCC were extracted as features.

#### 2.2.1. Short-Term Energy

The magnitude of STE effectively reflects the change law of the vocal signals with time and is representative [21,22]. Its calculation formula was

(1) $$E(n)=\sum_{-\infty }^{\infty }{\left[x(m)\omega (n-m)\right]}^{2}$$

where ***E***(***n***) was the short-time energy value when the window function was added at the ***n*th** energy point of the signal, ***x***(***m***) was the input pig vocal signal, ***m*** was the number of points at which the vocal signal was sampled, and ***ω***(***n***) was the selected window function.

**Figure 5:**
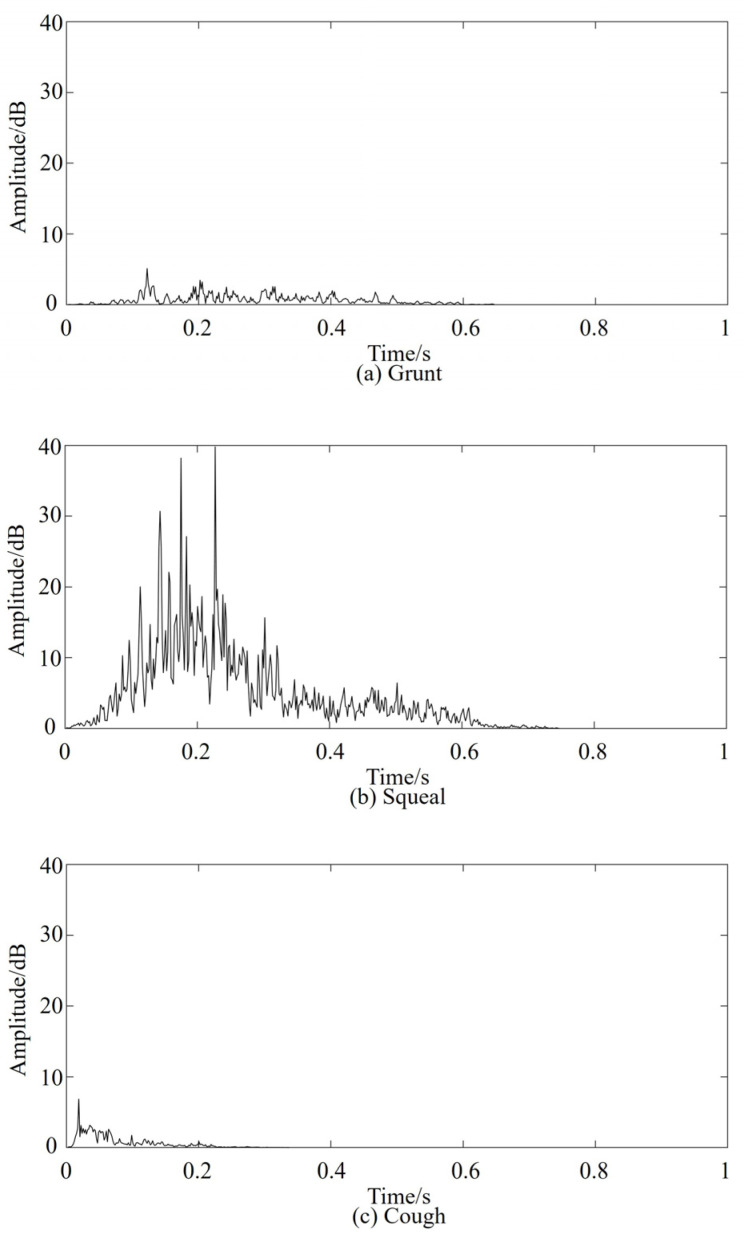
Short-term energy contrast diagram.

The STE of pig grunting, squealing, and coughing were calculated using vocal processing software. The features are shown in Figure 5.

#### 2.2.2. Frequency Centroid

The frequency centroid is an important component of the frequency and reflects the frequency mean of the voice based on the energy distribution. Supposing that the frequency centroid of the ***n*th** frame of {***x***(***m***)} was expressed as ***FC_n_***, the calculation formula was

(2) $$FC_{n}=\frac{\int_{0}^{\omega_{0}}\omega |X{\left(\omega \right)}^{2}|d\omega }{\int_{0}^{\omega_{0}}|X{\left(\omega \right)}^{2}|d\omega }.$$

Assuming that the total number of frames in {***x***(***m***)} was K and the frequency centroid distribution of the vocal signal in the frequency domain was expressed as the mean of the frequency centroid ***FC_avg_***, then the frequency centroid was defined as

(3) $$FC_{\mathrm{avg}}=\sqrt{\frac{1}{K}\sum_{n=1}^{K}FC_{n}}.$$

#### 2.2.3. Formant Frequency

**Figure 6:**
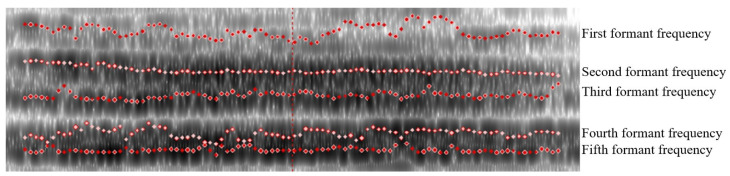
Formant frequency features of pig vocalization signals.

The formant frequency is a relatively concentrated area of energy in the vocal spectrum, which has a close relationship with the vocalization part and reflects the vocal quality features of the voice. The physical features of the vocal cavity can be inferred from the formant frequency parameters because the distribution ranges of the formant frequency parameters of different voices differ, and well distinguished different vocalizations [10]. Figure 6 shows the spectrogram of pig vocalization and reflects that there were obvious differences between different formant frequencies (from bottom to top, the first formant frequency, second formant frequency, third formant frequency, fourth formant frequency, and fifth formant frequency). The spectrogram reflected the initial examination of the data. Numerical data needed to be extracted to determine the differences between different vocalizations [10].

Vocal analysis software Praat (Version 6.2.1.6; developed by Boersma and Weenink, Institute of Phonetic Sciences, University of Amsterdam, Netherlands) was used to obtain the first formant frequency (FF-1), second formant frequency (FF-2), third formant frequency (FF-3), and fourth formant frequency (FF-4). The first-order difference ΔFF-21 was obtained from the difference between the second formant frequency and first formant frequency, the first-order difference ΔFF-32 was obtained from the difference between the third formant frequency and second formant frequency, and the first-order difference ΔFF-43 was obtained from the difference between the fourth formant frequency and third formant frequency.

#### 2.2.4. Mel Frequency Cepstral Coefficient

The MFCC was the typical acoustic feature to analyze the spectral features for pig cough recognition [23]. The MFCC had the advantages of simple calculation, good recognition performance, and strong noise immunity [18]. The calculation process was as follows:

(1) The original pig vocal signal ***S***(***n***) was pre-emphasized, framed, and added using the window function to obtain a time-domain signal ***X***(***n***).
(2) ***X***(***n***) was subjected to a fast Fourier transform to obtain a linear spectrum ***X***(***k***).
(3) ***X***(***k***) was filtered using the Mel bandpass triangle filter and logarithmic energy processing was conducted on the filtered signal to obtain the logarithmic spectrum of the acoustic signal ***S***(***m***).
(4) ***S***(***m***) was transformed to the cepstral domain using the discrete cosine transform and the MFCC was obtained.

**Figure 7:**
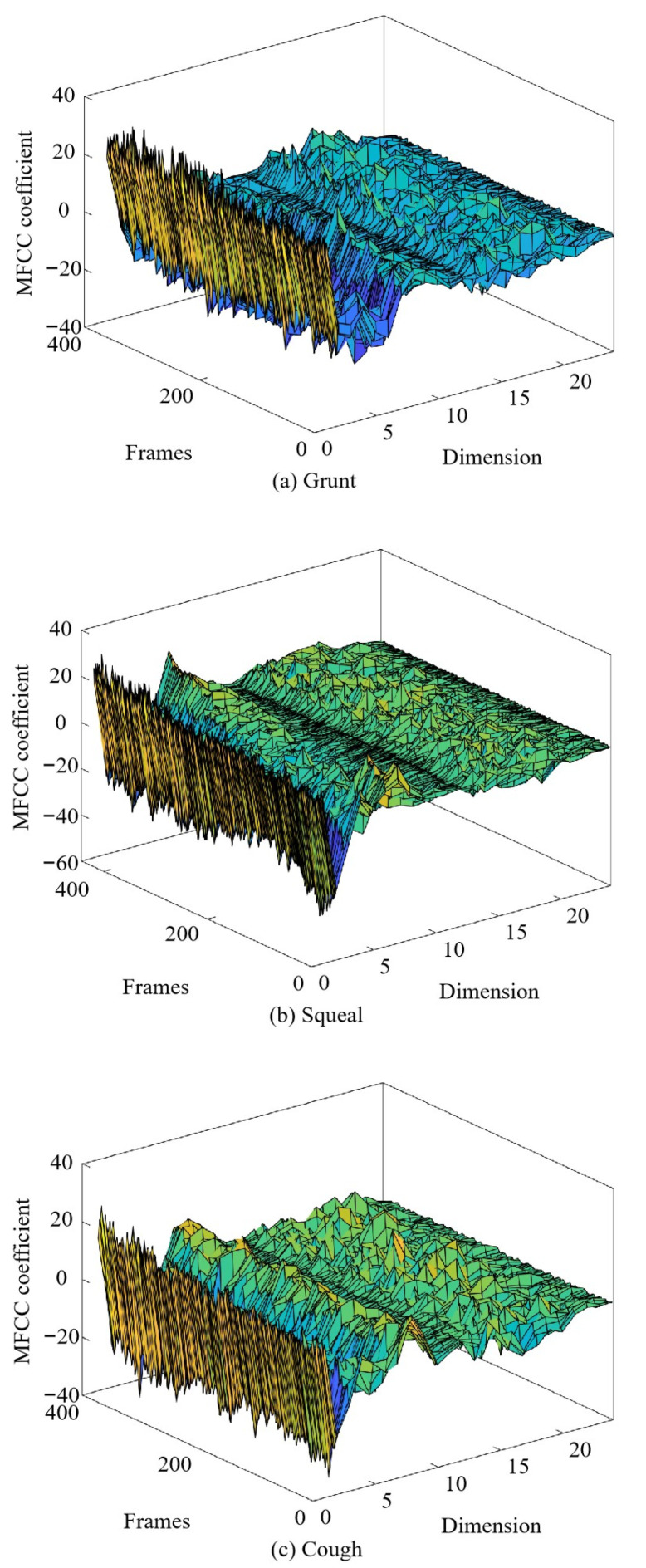
MFCC and first-order difference feature maps of various vocal signals of pigs.

The 2nd to 13th coefficients after the discrete cosine transformation in the aforementioned process were considered as the MFCC parameters to obtain a standard 12-dimensional MFCC parameter, which reflected the static features of the voice parameters. The dynamic features of the voice could be described by the difference spectrum of the static features; for example, the first-order difference characterized the speed of change of the vocal feature components and the second-order difference characterized the acceleration of change of the vocal feature components. Researchers have shown that the combination of the dynamic and static features of the MFCC effectively improved the parameter recognition performance. In this study, standard MFCC parameters and their first-order difference were combined to obtain 24-dimensional MFCC feature parameters, as shown in Figure 7.

### 2.3. Model Building

#### 2.3.1. Building a Comprehensive Evaluation Model for Pig Vocalization

PCA is a statistical method that converts a multi-indicator variable into a comprehensive indicator, which can effectively reduce the number of indicators to reduce the dimensionality of problems.

In this study, the main steps of PCA were as follows:

A total of 939 pig vocalization samples were obtained, each containing 33-dimensional features, based on which a variable matrix was established:

$$X=\left[\begin{array}{ccc} x_{1,1} & \cdots & x_{1,33}\\ \vdots & \ddots & \vdots \\ x_{939,1} & \cdots & x_{939,33} \end{array}\right]$$

where ***x_ij_***(***i***= 1,2,…,939; ***j***= 1,2,…,33) was the value of the ***j*th** dimension feature of the ***i*th** sample.

(1) To eliminate the numerical differences between sample factors, ensure the unity of dimensions, and simplify the data, it was necessary to standardize the data. The standardized data ***Z_ij_*** were obtained using the common standardization method, that is, the Z-Score transformation. The calculation formula was as follows:

(4) $$Z_{ij}=\frac{1}{S_{j}}(x_{ij}-\bar{x_{j}})$$

where $\bar{x_{j}}=\frac{1}{939}\sum_{i=1}^{939}x_{ij}$ was the mean of 939 variables and $S_{j}=\sqrt{\frac{1}{939-1}\sum_{i=1}^{939}{\left(x_{ij}-\bar{x_{j}}\right)}^{2}}$ was the mean square error of the ***j*th** factor.

(2) The correlation coefficient matrix R_33.33_ of the sample matrix was calculated and the elements in the matrix were calculated as follows:

(5) $$r_{ij}=\frac{1}{939-1}\sum_{i=1}^{939}x_{ti}x_{tj}\;i,j=1,2,\cdots ,33$$

(3) The eigenvalues of the correlation coefficient matrix and the corresponding eigenvectors were calculated, and the obtained eigenvectors formed the principal component coefficient matrix.

(4) The contribution rate and cumulative contribution rate of each component were calculated and the expression of the principal component was solved. The contribution rate ***C_i_*** was equal to the ratio of eigenvalues ***λ_i_*** corresponding to the principal component to the total eigenvalues, that is,

(6) $$C_{i}=\lambda /\sum_{i=1}^{33}\lambda_{i}$$

where the cumulative contribution rate of the first ***i*** principal components was ***δ_i_*** = ***C*_1_** + ***C*_2_** + … + ***C_i_***.

(5) The numerical features, such as the principal component load and principal component score, were calculated.

The principal component load was

(7) $$load=a_{i}\sqrt{\lambda_{i}}$$

where ***α_i_*** was the eigenvector corresponding to eigenvalue ***λ_i_***.

Starting from the original sample data matrix, the principal component score matrix was obtained after the principal component transformation.

(6) The principal component comprehensive evaluation score was calculated. Using the ratio of the variance contribution rate corresponding to each principal component to the cumulative contribution rate as the weight, linear weighting was performed on the principal component score and corresponding weight. Then, the comprehensive evaluation score was obtained.

Using the PCA method, the principal component score and total score of various vocalization types of each pig was obtained. In this study, the comprehensive evaluation score was introduced into the construction of the vocalization classification model as a new parameter for evaluating pig vocalization.

#### 2.3.2. Construction of the Pig Vocal Classification Model

**Figure 8:**
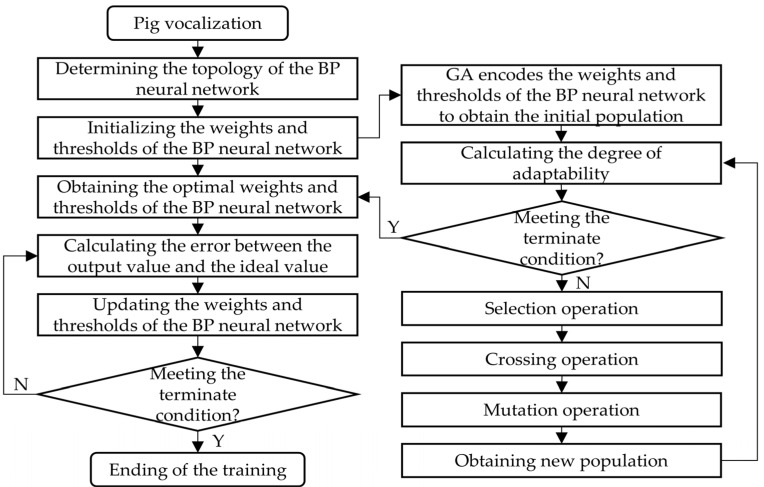
Flow chart of the BP neural network optimized using the genetic algorithm.

The BP neural network has been proved to be a useful way to classify abnormal pig sounds [24]. In this study, a typical three-layer BP neural network (input layer, hidden layer, and output layer) was selected to construct the pig vocalization classification model. The sigmoid function was used as the transfer function from the input layer to the hidden layer and from the hidden layer to the output layer. The number of nodes in the hidden layer was set to 10. The output layer was the recognition results of three types of vocal classification. The genetic algorithm was used to optimize the weights and thresholds of the neural network (GA-BP neural network) to improve the network training effect. Figure 8 shows the flow chart of the GA-BP neural network algorithm. As a result of the comparison of the effects of various group parameters on the recognition accuracy of the training set and validation set, the optimal value was selected. The number of training times was set to 1000, the target error was 0.00001, the learning rate was 0.01, the population size of the genetic algorithm was 50, the number of evolution times was 100, the crossover probability was 0.5, and the mutation probability was 0.01.

Using various feature combinations, a pig vocalization classification model was constructed and its classification recognition results were compared to optimize the model parameters. In the first group of tests, the short-time energy (1 dimension) of the time domain + frequency domain, spectral centroid (1 dimension), formant and first-order difference (7 dimensions), and MFCC and first-order difference (24 dimensions) were combined into 33-dimensional features as the input layer eigenvectors. In the second group, PCA was used to reduce the dimensionality of the 33-dimensional features. The 15-dimensional features of the 15 principal components generated when the cumulative contribution rate was greater than 85% were used as the input layer eigenvectors [25]. In the third group, a total of 16 dimensional features, including the 15 principal components obtained using PCA dimensionality reduction and the comprehensive principal component store, were used as the input layer eigenvectors. The number of input layer nodes in the three test groups was consistent with the dimensions of the input eigenvectors. In this study, GA-BP neural network models with three topologies (33–10–3, 15–10–3, and 16–10–3) were designed, and 70% of the test data were used for model training and 30% for model validation.

#### 2.3.3. Evaluation Indicators

To comprehensively evaluate the accuracy of the classification results, the three evaluation indicators of confusion matrix accuracy (***A***), precision (***P***), and recall (***R***) were used to compare the performance ability of each group of parameters [26]. Each indicator was calculated as follows:

(8) $$A\;=\frac{TP\;+TN}{TP\;+FN\;+TN\;+FP}$$

(9) $$P\;=\frac{TP}{TP\;+FP}$$

(10) $$R\;=\frac{TP}{TP\;+FN}$$

where ***TP*** was the positive class and judged to be the positive class, ***FP*** was the negative class and judged to be the positive class, ***FN*** was the positive class and judged to be the negative class, and ***TN*** was the negative class that was judged to be the negative class.

## 3. Results and Discussions

### 3.1. Analysis of the Features of Various Vocalization Types in Pigs

Table 1 shows the typical features of the three pig vocalization types. The duration of a squeal was significantly longer than that of a grunt and coughing (*p* < 0.05). The durations of grunts and coughs were 0.56 ± 0.15 s and 0.44 ± 0.07 s, respectively, and there was an intersection in the durations of the two types. There were significant differences in the STE of grunts, squeals, and coughs, as well as the frequency centroids and first, second, and third formant frequencies (*p* < 0.05). The difference between grunting and squealing for the fourth formant frequency was not significant (*p* < 0.05). Their average frequencies were 3855 ± 360 Hz and 3904 ± 259 Hz, respectively. For different types of pig vocalization, there were significant differences between their acoustic features to make it possible to use the features as important indicators for the digitization of their vocalization state. And this result might be a general characteristic for the three pig sound categories to help researchers understand pig sounds further, because this result was generated based on about 1000 sound samples.

### 3.2. Evaluation Scores of Various Types of Pig Vocalization Using Principal Component Analysis

**Figure 9:**
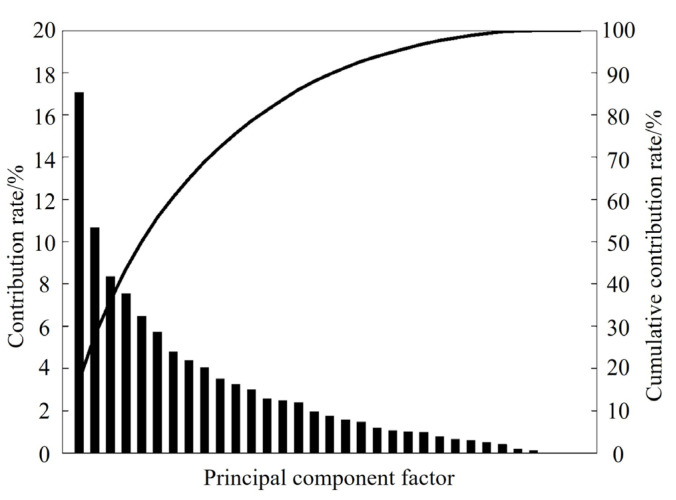
Principal components analysis results.

The short-time energy, frequency centroid, formant frequency and first-order difference, and MFCC and first-order difference parameters of grunting, squealing, and coughing samples were extracted, and their contribution rates were analyzed using PCA. Figure 9 shows the various contribution rates of each dimension of data in the 33-dimensional fusion features. According to the selection criteria, the first 15 components were selected as the principal components of pig acoustic features, because the cumulative contribution rates reached 85% [18]. The variance contribution rate of the first 15 principal components was 86.01%, which means that the first 15 principal components explained 86.01% of the original information, which indicated that the first 15 principal components interpreted the information of 33 features well [25].

The 15 principal components selected were denoted by ***F*_1_**, ***F*_2_**, …, ***F*_15_**, and the correlations between the 15 principal components and each feature were

(11) $$\{\begin{array}{c} F_{1}=0.26X_{1}+0.30X_{2}+\cdots +0.66X_{33}\\ F_{2}=-0.07X_{1}-0.05X_{2}+\cdots -0.17X_{33}\\ \vdots \\ F_{15}=0.31X_{1}-0.28X_{2}+\cdots +0.12X_{33} \end{array}$$

The eigenvalues corresponding to the 15 principal components were divided by the sum of the eigenvalues, and the quotient was used as the weight. Then, the comprehensive score function of the principal components was established:

(12) $$F_{\mathrm{comprehensive}}=0.20F_{1}+0.12F_{2}+\cdots +0.02F_{15}$$

The scores are shown in Table 2.

**Figure 10:**
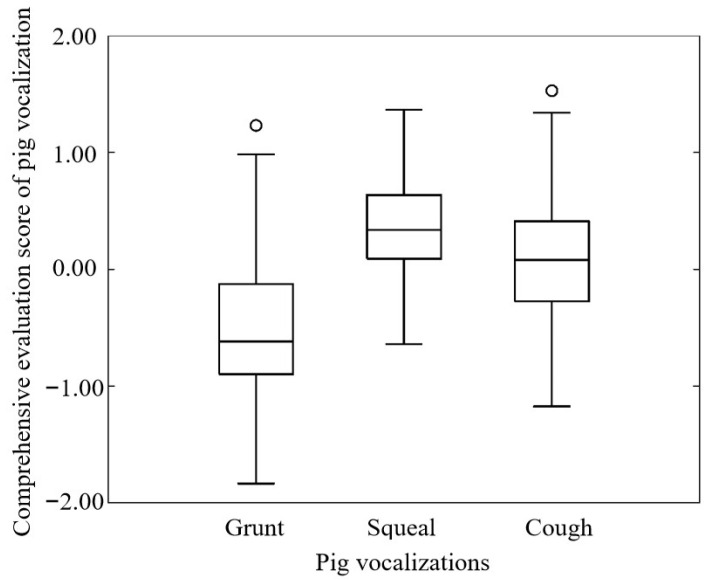
Box plot of the comprehensive evaluation score of pig vocalization.

To better illustrate the differences between different types of pig vocalization, a box plot was drawn using the comprehensive evaluation scores of various vocal samples of pigs. As shown in Figure 10, the average comprehensive evaluation score of pig grunting was −0.52 and the distribution range of the comprehensive evaluation score was −1.07~0.03; the average comprehensive evaluation score of pig squealing was 0.35 and the distribution range of the comprehensive evaluation score was −0.03~0.73; the average comprehensive evaluation score of pig coughing was 0.08 and the distribution range of the comprehensive evaluation score was −0.42~0.58. There were significant differences in the comprehensive evaluation score of pig vocalization between the groups (*p* < 0.001), which indicated that the comprehensive evaluation score could be used as an effective indicator for the classification and recognition of various types of pig vocalization.

### 3.3. Comparative Analysis of the Recognition Effects of Various Types of Pig Vocalization

The typical time frequency domain features of pig vocal signals were taken into account in this study. Table 3, Table 4 and Table 5 show that the recall of squealing was higher than that of grunting and coughing, which was mainly because the spectral features of squealing were more significant and it was not prone to be confused with other types of pig vocalization. By contrast, the vocal intensity of grunting was low, its feature change was not obvious from coughing, and vocal syncopation was prone to be affected by background noise and human interference [20].

Table 6 showed that the recognition accuracy (93.2%) of the vocalization classification model with 16-dimensional features based on the “15 principal components + comprehensive evaluation score” was slightly higher than that of the model with “15 principal components” (91.5%) and that with “STE + FC + FF + ΔFF + MFCC + ΔMFCC” as the fusion feature (90.7%) based on different time-frequency domain feature combinations and parameter dimensions. Although the features of the fused STE + FC + FF + ΔFF + MFCC + ΔMFCC described the original vocal data from multiple aspects, there were still a large number of redundant components, which affected the classification and recognition effect of the model [14,20]. After PCA dimensionality reduction, the vocalization classification model’s classification and recognition accuracies for various types of pig vocalization improved, which illustrated that effective dimensionality reduction could reduce redundant components and improve the efficiency of useful features extracting [18]. Additionally, dimensionality reduction greatly reduced model computation and improved the working performance of the model.

The vocalization classification model constructed by introducing the comprehensive evaluation score had the best performance in terms of classification recognition and model operation efficiency, with recognition precisions of 87.9%, 98.1%, and 92.7% and recognition recalls of 92.0%, 99.1%, and 87.4% for pig grunting, squealing, and coughing, respectively. The comprehensive evaluation scores of the three types of pig vocalization were significantly different (Figure 10), which indicated that the vocalization classification model’s ability to recognize various types of pig vocalization was effectively improved by the introduction of features that could distinguish the types of pig vocalization clearly. It was also supporting that comprehensive evaluation score could be used as the useful and important acoustic feature in animal vocalization classification. Additionally, Yin et al. [8] also found that exploiting inter-feature variability can compensate for the limitations of a single feature, and model accuracy failed to improve and remained stable as more features were added, which supported our results and proved the comprehensive evaluation score was an efficient feature for pig sound classification. In recent studies for pig cough recognition, their classification accuracies could achieve above 95% [7,8,14], which were higher than that of our study (93.2%). Because all of these studies carried out binary classification tasks with deep learning algorithms, while it was a three-class classification task with a simple deep learning algorithm in our study. The more classification tasks, the more complex the calculation process of the model is. On the basis of maintaining similar results to peer research in cough recognition, our model could also maintain high classification accuracies for grunts and squeals, indicating the feasibility and efficiency of using the multi-feature fusion algorithm for pig vocalization classification. In addition, compared to complex neural networks, a shallow neural network offers a definite advantage in terms of processing time [7]. Zhuang et al. [27] also proved that a lightweight model could achieve good performance with a shorter training duration, simpler training process, and less computility, which was more suitable for application in livestock and poultry farms.

Sound is one of the important pieces of physical information of pigs, so it is significant to use the information contained in the sound of pigs to determine the current status of pigs [19]. The classification model developed in this study could be applied as a warning tool or/and supplementary method in assessing air quality inside livestock buildings, especially around the animal occupied zone, to facilitate efficient management and precision livestock farming. Limited by field conditions, only one acoustic test analyzer was placed in a pig house to inevitably make data quality impacted by the distance [28]. Consequently, the classification accuracy may degrade in real-life production to some extent, but in general, its working performance would not be significantly affected. It is suggested to add more acoustic test equipment to improve the acoustic data quality in future studies [7].

## 4. Conclusions

In this study, a pig vocalization classification recognition method was proposed based on the GA-BP neural network and multi-feature fusion with the time domain, frequency domain, and comprehensive evaluation score. The classification recognition of pig grunting, squealing, and coughing was performed, and then the recognition performances of classification models with various feature combinations for various types of pig vocalization were compared and optimized. After the dimensionality of short-time energy, frequency centroid, formant frequency and first-order difference, and MFCC and first-order difference feature were reduced using PCA, the vocalization classification model constructed using the 16-dimensional features, which included the comprehensive evaluation score of pig vocalization, had the highest recognition performance for three types of pig vocalization, with an average recognition accuracy of 93.2%, average precision of 92.9%, and average recall of 92.8%. It was feasible and efficient to apply the multi-feature fusion algorithm to the classification of pig vocalization, and the introduction of features that clearly distinguished vocalization types effectively improved the recognition ability of the vocalization classification model for various types of pig vocalization.

## Figures and Tables

**Table 1:** Statistics and analysis of the typical features of pig vocalizations.

	Samples	Duration	STE	FC	FF-1	FF-2	FF-3	FF-4
Grunt	291	0.56	0.48	1316.96	893.73	1608.20	3014.85	3854.69
		±	±	±	±	±	±	±
		0.15 a	0.25 a	300.06 a	145.58 a	454.76 a	368.39 a	359.54 a
Squeal	357	1.01	4.14	1833.87	1158.01	1924.88	2898.51	3903.99
		±	±	±	±	±	±	±
		0.35 b	2.75 b	433.17 b	193.06 b	283.97 b	243.76 b	258.53 a
Cough	291	0.44	0.69	1400.75	964.28	1723.21	2846.6	3796.09
		±	±	±	±	±	±	±
		0.07 c	0.49 c	271.26 c	132.88 c	328.3 c	311.94 c	286.01 b

Note: Different letters in the same column mean a significant difference at the 0.05 level.

**Table 2:** Comprehensive scores of various vocal signals of pigs.

Vocal Signal No.	*F*_1_	*F*_2_	⋯	*F*_15_	*F*_Comprehensive_
1	−3.56	2.33	⋯	−0.25	−1.05
2	−3.48	−3.19	⋯	−0.29	−1.23
⋯	⋯	⋯	⋯	⋯	⋯
939	−3.63	−2.17	⋯	1.81	−0.64

**Table 3:** Confusion matrix of pig vocalization classification and recognition based on STE + FC + FF + ΔFF + MFCC + ΔMFCC.

Type of Vocalization	Grunt	Squeal	Cough	Total	Recall/%
Grunt	77	3	7	87	88.5
Squeal	1	106	0	107	99.1
Cough	14	1	72	87	82.8
Total	92	110	79		
Precision/%	83.7	96.4	91.1		

**Table 4:** Confusion matrix of pig vocalization classification and recognition based on 15 principal components.

Type of Vocalization	Grunt	Squeal	Cough	Total	Recall/%
Grunt	78	0	9	87	89.7
Squeal	1	106	0	107	99.1
Cough	12	2	73	87	83.9
Total	91	108	82		
Precision/%	85.7	98.1	89.0		

**Table 5:** Confusion matrix for the vocalization classification and recognition of pigs based on 15 principal components and comprehensive evaluation scores.

Type of Vocalization	Grunt	Squeal	Cough	Total	Recall/%
Grunt	80	1	6	87	92.0
Squeal	1	106	0	107	99.1
Cough	10	1	76	87	87.4
Total	91	108	82		
Precision/%	87.9	98.1	92.7		

**Table 6:** Comparison of the classification and recognition performance of pig vocalization.

Feature Parameters	Dimension	Average Recognition Accuracy/%
STE + FC + FF + ΔFF + MFCC + ΔMFCC	33	90.7
15 principal components	15	91.5
15 principal components + comprehensive evaluation score	16	93.2

## Data Availability

The data presented in this study are available on request from the corresponding author.
